# 3-Methyl-1,4-diphenyl-1*H*-pyrazolo­[3,4-*b*]quinoline

**DOI:** 10.1107/S1600536810042935

**Published:** 2010-10-31

**Authors:** Paweł Szlachcic, Andrzej Danel, Katarzyna Stadnicka

**Affiliations:** a Department of Chemistry and Physics, Agricultural University, 30-149 Kraków, Poland; b Faculty of Chemistry, Jagiellonian University, 30-060 Kraków, Poland

## Abstract

In the title mol­ecule, C_23_H_17_N_3_, the phenyl substituents at positions 1 and 4 are twisted relative to the central core by 27.09 (5) and 66.62 (4)°, respectively. In the crystal, mol­ecules are assembled into centrosymmetric dimers *via* π–π stacking inter­actions between the 1*H*-pyrazolo­[3,4-*b*]quinoline ­units, with an inter­planar distance of 3.601 (2) Å and by weak inter­molecular C—H⋯N inter­actions.

## Related literature

For the synthesis of 1,3 and 4-substituted 1*H*-pyrazolo­[3,4-*b*]quinoline derivatives using Friedländer condensation, see: Danel (1996 ▶); Woo *et al.* (2002 ▶). For selected photophysical properties of 1*H*-pyrazolo­[3,4-*b*]quinoline derivatives, see: Gondek *et al.* (2006 ▶). For related structures, see: Szlachcic & Stadnicka (2010 ▶); Szlachcic *et al.* (2010 ▶).
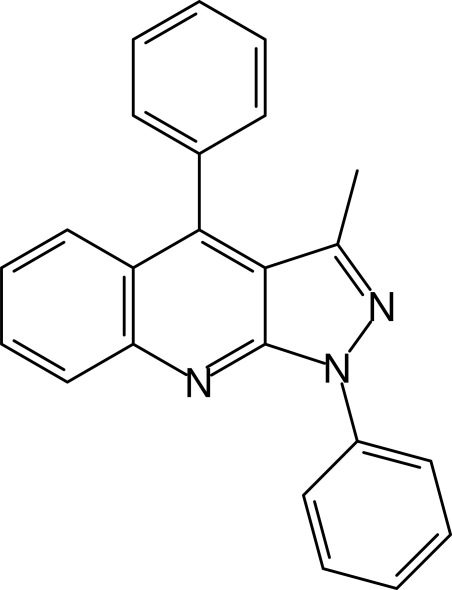


## Experimental

### 

#### Crystal data

C_23_H_17_N_3_
*M* *_r_* = 335.40
Triclinic, 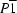

*a* = 9.2120 (4) Å
*b* = 9.9377 (5) Å
*c* = 10.3440 (4) Å
α = 92.278 (2)°
β = 113.376 (2)°
γ = 90.152 (2)°
*V* = 868.37 (7) Å^3^
*Z* = 2
Mo *K*α radiation
μ = 0.08 mm^−1^
*T* = 293 K
0.50 × 0.42 × 0.15 mm

#### Data collection

Nonius KappaCCD diffractometer
Absorption correction: multi-scan (*DENZO* and *SCALEPACK*; Otwinowski & Minor, 1997 ▶) *T*_min_ = 0.963, *T*_max_ = 0.989
6556 measured reflections
4964 independent reflections
3285 reflections with *I* > 2σ(*I*)
*R*_int_ = 0.020

#### Refinement

*R*[*F* ^2^ > 2σ(*F* ^2^)] = 0.053
*wR*(*F* ^2^) = 0.139
*S* = 1.02
4964 reflections
236 parameters
H-atom parameters constrained
Δρ_max_ = 0.24 e Å^−3^
Δρ_min_ = −0.17 e Å^−3^

### 

Data collection: *COLLECT* (Nonius, 1998 ▶); cell refinement: *SCALEPACK* (Otwinowski & Minor, 1997 ▶); data reduction: *DENZO* (Otwinowski & Minor, 1997 ▶) and *SCALEPACK*; program(s) used to solve structure: *SIR92* (Altomare *et al.*, 1994 ▶); program(s) used to refine structure: *SHELXL97* (Sheldrick, 2008 ▶); molecular graphics: *ORTEP-3* (Farrugia, 1997 ▶); software used to prepare material for publication: *SHELXL97*.

## Supplementary Material

Crystal structure: contains datablocks global, I. DOI: 10.1107/S1600536810042935/gk2306sup1.cif

Structure factors: contains datablocks I. DOI: 10.1107/S1600536810042935/gk2306Isup2.hkl

Additional supplementary materials:  crystallographic information; 3D view; checkCIF report

## Figures and Tables

**Table 1:** Hydrogen-bond geometry (Å, °)

*D*—H⋯*A*	*D*—H	H⋯*A*	*D*⋯*A*	*D*—H⋯*A*
C46—H46⋯N9^i^	0.93	2.52	3.4164 (18)	163

Symmetry code: (i) 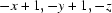.

**Fig. 1.**
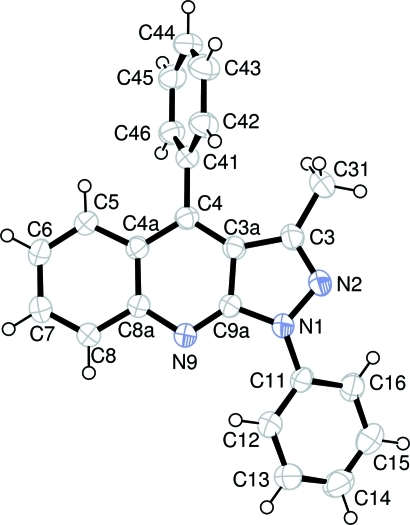
The conformation of the 3-methyl-1,4-diphenyl-1H-pyrazolo[3,4-b]quinoline molecule with the atom numbering scheme. Displacement ellipsoids are drawn at the 50% probability level and H atoms are shown as small spheres of arbitrary radii.

**Fig. 2.**
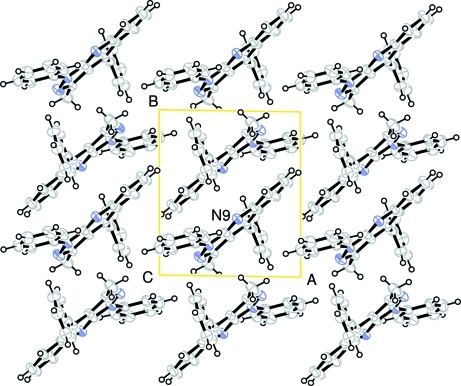
The unit-cell contents of the title compound in projection along [001] showing molecular dimers formation.

## supplementary crystallographic information

### Comment

The title compound and other 1*H*-pyrazolo[3,4-*b*]quinoline (PQ) derivatives containing hydrogen, methyl or phenyl substituents and their combination, showed important photophysical properties (Gondek *et al.*, 2006) which could be utilized in organic light-emitting diodes (OLED) fabrication. To synthesize 1,3,4-substituted PQ derivatives, a method of preparation introduced by Danel (1996) was used. The results of using the title compound in OLED preparation will be published elsewhere.

The shape of the title molecule is shown in Fig. 1. The core of the molecule, 1*H*-pyrazolo[3,4-*b*]quinoline, is planar and aromatic. The planes of phenyl substituents at positions 1 and 4 are twisted against the core moiety with the torsion angles N2—N1—C11—C16 = -15.7 (2) and C3a—C4—C41—C46 = 116.7 (2)°. The conformation of the molecule is stabilized by two intramolecular interactions of C—H···N type in which N2 and N9 atoms are acceptors.

The packing of the molecules (Fig. 2) is determined by one weak intermolecular hydrogen bond C46—H46···N9 (-*x* + 1, -*y* + 1, -*z*), and π-π interactions: with *Cg*1 (N1—N2—C3—C3a—C9a)···*Cg*3 (C4a—C5—C6—C7—C8—C8a at 1 - *x*, 1 - *y*, -*z*) = 3.731 and *Cg*2 (C3a—C4—C4a—C8a—N9—C9a)···*Cg*2 (C3a—C4—C4a—C8a—N9—C9a at 1 - *x*, 1 - *y*, -*z*) = 3.799 Å resulting in forming molecular dimers. The two C—H···π interactions are described by the geometry parameters (H···A /Å, D···A /Å, <DHA /°, respectively) given below:

C6—H6···*Cg*5 (C41—C42—C43—C44—C45—C46 at 2 - *x*, 1 - *y*, -*z*): 2.967, 3.750, 143;

C31—H31···*Cg*1 (N1—N2—C3—C3a—C9a at 1 - *x*, -*y*, -*z*): 3.172, 3.875, 132.

### Experimental

The title compound was synthesized using procedure already described in literature (Danel, 1996) from 2-aminobenzophenone and 5-methyl-2-phenyl-2,4-dihydro-pyrazol-3-one (10 mmol of each substrate, ethylene glycol as a solvent). The product was purified by flash chromatography on Al_2_O_3_ with chloroform as a solvent, followed by crystallization from toluene/petroleum ether to give 2.38 g (71% yield) of light-yellow crystalline solid, mp. 438–440 K. ^1^H NMR (CDCl_3_): δ 2.14 (s, 3H), 7.25–7.30 (m, 1H), 7.36 (ddd, *J* = 8.6, 6.7, 1.3 Hz, 1H), 7.44–7.47 (m, 2H), 7.52–7.60 (m, 5H). 7.71–7.77 (m, 2H), 8.20 (d, *J* = 8.4 Hz, 1H), 8.49–8.53 (m, 2H). ^13^C NMR (CDCl_3_): δ 14.9, 116.3, 120.3, 123.6, 123.9, 124.9, 127.0, 128.3, 128.7, 129.0 (two signals), 129.7, 130.3, 135.0, 140.0, 143.8, 144.4, 148.5, 150.2. Single crystals suitable for X-ray diffraction were grown by slow evaporation from toluene solution at ambient conditions.

### Refinement

H atoms were included into refinement in geometrically calculated positions, with C—H = 0.93 Å and *U*_iso_(H) = 1.2*U*_eq_ for the aromatic CH groups and C—H = 0.96 Å and *U*_iso_(H) = 1.5*U*_eq_ for methyl groups. The positions of H atoms were constrained as a part of a riding model. In the case of methyl group the torsion angle along the C_aromatic_—C_methyl_ bond was refined using AFIX 137 procedure (*SHELXL-97*; Sheldrick, 2008).

### Crystal data

**Table:**

C_23_H_17_N_3_	*Z* = 2
*M**_r_* = 335.40	*F*(000) = 352
Triclinic, *P*1	*D*_x_ = 1.283 Mg m^−^^3^
Hall symbol: -P 1	Melting point = 438–440 K
*a* = 9.2120 (4) Å	Mo *K*α radiation, λ = 0.71073 Å
*b* = 9.9377 (5) Å	Cell parameters from 2458 reflections
*c* = 10.3440 (4) Å	θ = 1.0–30.0°
α = 92.278 (2)°	µ = 0.08 mm^−^^1^
β = 113.376 (2)°	*T* = 293 K
γ = 90.152 (2)°	Plate, green–yellow
*V* = 868.37 (7) Å^3^	0.50 × 0.42 × 0.15 mm

### Data collection

**Table:**

Nonius KappaCCD diffractometer	4964 independent reflections
Radiation source: fine-focus sealed tube	3285 reflections with *I* > 2σ(*I*)
horizontally mounted graphite crystal	*R*_int_ = 0.020
Detector resolution: 9 pixels mm^-1^	θ_max_ = 30.0°, θ_min_ = 2.9°
φ and ο scans to fill Ewald sphere	*h* = −12→11
Absorption correction: multi-scan (*DENZO* and *SCALEPACK*; Otwinowski & Minor, 1997)	*k* = −13→7
*T*_min_ = 0.963, *T*_max_ = 0.989	*l* = −14→13
6556 measured reflections	

### Refinement

**Table:**

Refinement on *F*^2^	Primary atom site location: structure-invariant direct methods
Least-squares matrix: full	Secondary atom site location: difference Fourier map
*R*[*F*^2^ > 2σ(*F*^2^)] = 0.053	Hydrogen site location: inferred from neighbouring sites
*wR*(*F*^2^) = 0.139	H-atom parameters constrained
*S* = 1.02	*w* = 1/[σ^2^(*F*_o_^2^) + (0.054*P*)^2^ + 0.1697*P*] where *P* = (*F*_o_^2^ + 2*F*_c_^2^)/3
4964 reflections	(Δ/σ)_max_ < 0.001
236 parameters	Δρ_max_ = 0.24 e Å^−^^3^
0 restraints	Δρ_min_ = −0.17 e Å^−^^3^
0 constraints	

### Special details

**Table:**

Geometry. All e.s.d.'s (except the e.s.d. in the dihedral angle between two l.s. planes) are estimated using the full covariance matrix. The cell e.s.d.'s are taken into account individually in the estimation of e.s.d.'s in distances, angles and torsion angles; correlations between e.s.d.'s in cell parameters are only used when they are defined by crystal symmetry. An approximate (isotropic) treatment of cell e.s.d.'s is used for estimating e.s.d.'s involving l.s. planes.

### Fractional atomic coordinates and isotropic or equivalent isotropic displacement parameters (Å^2^)

**Table:**

	*x*	*y*	*z*	*U*_iso_*/*U*_eq_	
N1	0.34388 (14)	0.18687 (11)	0.08010 (12)	0.0397 (3)	
N2	0.28625 (15)	0.11194 (12)	−0.04633 (13)	0.0438 (3)	
C3	0.37588 (17)	0.13841 (14)	−0.11453 (15)	0.0416 (3)	
C3A	0.49700 (16)	0.23706 (13)	−0.03567 (14)	0.0351 (3)	
C4	0.61386 (15)	0.31001 (13)	−0.05685 (14)	0.0344 (3)	
C4A	0.70159 (15)	0.40697 (13)	0.05158 (14)	0.0345 (3)	
C5	0.82339 (17)	0.49062 (14)	0.04416 (15)	0.0418 (3)	
H5	0.8450	0.4851	−0.0364	0.050*	
C6	0.90904 (18)	0.57853 (16)	0.15214 (17)	0.0478 (4)	
H6	0.9870	0.6330	0.1441	0.057*	
C7	0.88014 (19)	0.58731 (16)	0.27575 (17)	0.0514 (4)	
H7	0.9402	0.6466	0.3497	0.062*	
C8	0.76495 (18)	0.50976 (15)	0.28780 (16)	0.0464 (4)	
H8	0.7476	0.5163	0.3704	0.056*	
C8A	0.67061 (15)	0.41895 (13)	0.17655 (14)	0.0356 (3)	
N9	0.55506 (13)	0.34765 (11)	0.19637 (12)	0.0381 (3)	
C9A	0.47232 (15)	0.26467 (13)	0.08992 (14)	0.0343 (3)	
C11	0.25132 (16)	0.19574 (13)	0.16186 (14)	0.0378 (3)	
C12	0.31890 (19)	0.24076 (15)	0.30166 (16)	0.0487 (4)	
H12	0.4260	0.2639	0.3436	0.058*	
C13	0.2259 (2)	0.25117 (17)	0.37876 (19)	0.0579 (4)	
H13	0.2706	0.2831	0.4723	0.069*	
C14	0.0681 (2)	0.21477 (17)	0.3187 (2)	0.0591 (4)	
H14	0.0064	0.2220	0.3712	0.071*	
C15	0.00234 (19)	0.16761 (17)	0.18044 (19)	0.0553 (4)	
H15	−0.1038	0.1413	0.1401	0.066*	
C16	0.09222 (17)	0.15891 (15)	0.10080 (17)	0.0455 (3)	
H16	0.0464	0.1285	0.0068	0.055*	
C31	0.3400 (2)	0.06994 (18)	−0.25516 (17)	0.0578 (4)	
H31A	0.4209	0.0063	−0.2477	0.087*	
H31B	0.3365	0.1358	−0.3217	0.087*	
H31C	0.2395	0.0237	−0.2866	0.087*	
C41	0.64981 (16)	0.28577 (13)	−0.18381 (14)	0.0357 (3)	
C42	0.70864 (19)	0.16239 (15)	−0.20626 (16)	0.0462 (4)	
H42	0.7243	0.0953	−0.1421	0.055*	
C43	0.7441 (2)	0.13829 (17)	−0.32315 (18)	0.0540 (4)	
H43	0.7836	0.0555	−0.3371	0.065*	
C44	0.72084 (19)	0.23688 (18)	−0.41857 (17)	0.0524 (4)	
H44	0.7443	0.2207	−0.4973	0.063*	
C45	0.66269 (18)	0.35998 (17)	−0.39743 (16)	0.0491 (4)	
H45	0.6468	0.4265	−0.4622	0.059*	
C46	0.62795 (17)	0.38491 (14)	−0.28066 (15)	0.0417 (3)	
H46	0.5898	0.4684	−0.2667	0.050*	

### Atomic displacement parameters (Å^2^)

**Table:**

	*U*^11^	*U*^22^	*U*^33^	*U*^12^	*U*^13^	*U*^23^
N1	0.0428 (6)	0.0404 (6)	0.0370 (6)	−0.0120 (5)	0.0179 (5)	−0.0042 (5)
N2	0.0489 (7)	0.0417 (6)	0.0396 (6)	−0.0131 (5)	0.0172 (5)	−0.0058 (5)
C3	0.0461 (8)	0.0386 (7)	0.0390 (7)	−0.0073 (6)	0.0164 (6)	−0.0017 (6)
C3A	0.0399 (7)	0.0325 (6)	0.0335 (6)	−0.0019 (5)	0.0153 (6)	0.0008 (5)
C4	0.0363 (7)	0.0329 (6)	0.0353 (7)	0.0014 (5)	0.0156 (6)	0.0018 (5)
C4A	0.0342 (7)	0.0335 (6)	0.0375 (7)	−0.0004 (5)	0.0162 (6)	−0.0003 (5)
C5	0.0409 (8)	0.0449 (8)	0.0444 (8)	−0.0061 (6)	0.0223 (6)	−0.0029 (6)
C6	0.0417 (8)	0.0495 (8)	0.0561 (9)	−0.0134 (6)	0.0243 (7)	−0.0065 (7)
C7	0.0485 (9)	0.0547 (9)	0.0517 (9)	−0.0176 (7)	0.0225 (7)	−0.0156 (7)
C8	0.0461 (8)	0.0537 (8)	0.0429 (8)	−0.0131 (7)	0.0230 (7)	−0.0122 (7)
C8A	0.0351 (7)	0.0359 (6)	0.0372 (7)	−0.0031 (5)	0.0160 (6)	−0.0012 (5)
N9	0.0389 (6)	0.0401 (6)	0.0370 (6)	−0.0073 (5)	0.0173 (5)	−0.0032 (5)
C9A	0.0359 (7)	0.0331 (6)	0.0352 (6)	−0.0033 (5)	0.0155 (5)	0.0018 (5)
C11	0.0420 (7)	0.0326 (6)	0.0419 (7)	−0.0047 (5)	0.0198 (6)	0.0043 (6)
C12	0.0521 (9)	0.0529 (9)	0.0428 (8)	−0.0151 (7)	0.0210 (7)	−0.0023 (7)
C13	0.0749 (12)	0.0548 (9)	0.0531 (10)	−0.0144 (8)	0.0360 (9)	−0.0073 (8)
C14	0.0658 (11)	0.0563 (10)	0.0722 (12)	0.0003 (8)	0.0455 (10)	0.0011 (9)
C15	0.0425 (9)	0.0590 (10)	0.0689 (11)	−0.0006 (7)	0.0265 (8)	0.0083 (9)
C16	0.0411 (8)	0.0467 (8)	0.0467 (8)	−0.0052 (6)	0.0151 (7)	0.0040 (7)
C31	0.0659 (11)	0.0612 (10)	0.0460 (9)	−0.0213 (8)	0.0237 (8)	−0.0155 (8)
C41	0.0369 (7)	0.0365 (7)	0.0355 (7)	−0.0032 (5)	0.0167 (6)	−0.0012 (6)
C42	0.0563 (9)	0.0398 (7)	0.0454 (8)	0.0040 (6)	0.0235 (7)	0.0007 (6)
C43	0.0632 (10)	0.0498 (9)	0.0546 (10)	0.0061 (8)	0.0301 (8)	−0.0069 (8)
C44	0.0532 (9)	0.0675 (10)	0.0417 (8)	−0.0034 (8)	0.0256 (7)	−0.0085 (8)
C45	0.0523 (9)	0.0582 (9)	0.0403 (8)	−0.0035 (7)	0.0218 (7)	0.0067 (7)
C46	0.0453 (8)	0.0412 (7)	0.0421 (8)	0.0010 (6)	0.0212 (6)	0.0029 (6)

### Geometric parameters (Å, °)

**Table:**

N1—C9A	1.3790 (16)	C12—C13	1.385 (2)
N1—N2	1.3842 (16)	C12—H12	0.9300
N1—C11	1.4201 (17)	C13—C14	1.376 (3)
N2—C3	1.3132 (18)	C13—H13	0.9300
C3—C3A	1.4422 (19)	C14—C15	1.374 (3)
C3—C31	1.492 (2)	C14—H14	0.9300
C3A—C4	1.3885 (18)	C15—C16	1.381 (2)
C3A—C9A	1.4217 (18)	C15—H15	0.9300
C4—C4A	1.4249 (18)	C16—H16	0.9300
C4—C41	1.4876 (18)	C31—H31A	0.9600
C4A—C5	1.4228 (18)	C31—H31B	0.9600
C4A—C8A	1.4308 (18)	C31—H31C	0.9600
C5—C6	1.361 (2)	C41—C42	1.3898 (19)
C5—H5	0.9300	C41—C46	1.3907 (19)
C6—C7	1.405 (2)	C42—C43	1.384 (2)
C6—H6	0.9300	C42—H42	0.9300
C7—C8	1.358 (2)	C43—C44	1.375 (2)
C7—H7	0.9300	C43—H43	0.9300
C8—C8A	1.4190 (19)	C44—C45	1.381 (2)
C8—H8	0.9300	C44—H44	0.9300
C8A—N9	1.3631 (16)	C45—C46	1.381 (2)
N9—C9A	1.3160 (17)	C45—H45	0.9300
C11—C12	1.383 (2)	C46—H46	0.9300
C11—C16	1.3875 (19)		
			
C9A—N1—N2	110.06 (11)	C11—C12—H12	120.3
C9A—N1—C11	129.37 (11)	C13—C12—H12	120.3
N2—N1—C11	119.07 (11)	C14—C13—C12	120.78 (16)
C3—N2—N1	108.04 (11)	C14—C13—H13	119.6
N2—C3—C3A	110.55 (12)	C12—C13—H13	119.6
N2—C3—C31	119.20 (13)	C15—C14—C13	119.47 (15)
C3A—C3—C31	130.23 (13)	C15—C14—H14	120.3
C4—C3A—C9A	118.45 (12)	C13—C14—H14	120.3
C4—C3A—C3	136.89 (13)	C14—C15—C16	120.71 (16)
C9A—C3A—C3	104.51 (11)	C14—C15—H15	119.6
C3A—C4—C4A	116.60 (12)	C16—C15—H15	119.6
C3A—C4—C41	122.02 (12)	C15—C16—C11	119.61 (15)
C4A—C4—C41	121.36 (11)	C15—C16—H16	120.2
C5—C4A—C4	123.14 (12)	C11—C16—H16	120.2
C5—C4A—C8A	117.71 (12)	C3—C31—H31A	109.5
C4—C4A—C8A	119.12 (11)	C3—C31—H31B	109.5
C6—C5—C4A	121.57 (13)	H31A—C31—H31B	109.5
C6—C5—H5	119.2	C3—C31—H31C	109.5
C4A—C5—H5	119.2	H31A—C31—H31C	109.5
C5—C6—C7	120.27 (13)	H31B—C31—H31C	109.5
C5—C6—H6	119.9	C42—C41—C46	118.74 (12)
C7—C6—H6	119.9	C42—C41—C4	119.81 (12)
C8—C7—C6	120.36 (14)	C46—C41—C4	121.45 (12)
C8—C7—H7	119.8	C43—C42—C41	120.68 (14)
C6—C7—H7	119.8	C43—C42—H42	119.7
C7—C8—C8A	121.18 (14)	C41—C42—H42	119.7
C7—C8—H8	119.4	C44—C43—C42	119.96 (14)
C8A—C8—H8	119.4	C44—C43—H43	120.0
N9—C8A—C8	117.20 (12)	C42—C43—H43	120.0
N9—C8A—C4A	123.94 (12)	C43—C44—C45	119.95 (14)
C8—C8A—C4A	118.86 (12)	C43—C44—H44	120.0
C9A—N9—C8A	114.32 (11)	C45—C44—H44	120.0
N9—C9A—N1	125.83 (12)	C46—C45—C44	120.36 (14)
N9—C9A—C3A	127.35 (12)	C46—C45—H45	119.8
N1—C9A—C3A	106.81 (11)	C44—C45—H45	119.8
C12—C11—C16	119.95 (13)	C45—C46—C41	120.30 (13)
C12—C11—N1	120.37 (13)	C45—C46—H46	119.9
C16—C11—N1	119.67 (13)	C41—C46—H46	119.9
C11—C12—C13	119.46 (15)		
			
C9A—N1—N2—C3	1.12 (16)	C11—N1—C9A—N9	14.3 (2)
C11—N1—N2—C3	168.47 (12)	N2—N1—C9A—C3A	−0.24 (15)
N1—N2—C3—C3A	−1.54 (16)	C11—N1—C9A—C3A	−165.91 (13)
N1—N2—C3—C31	179.75 (13)	C4—C3A—C9A—N9	−4.6 (2)
N2—C3—C3A—C4	−173.86 (15)	C3—C3A—C9A—N9	179.13 (13)
C31—C3—C3A—C4	4.7 (3)	C4—C3A—C9A—N1	175.65 (12)
N2—C3—C3A—C9A	1.37 (16)	C3—C3A—C9A—N1	−0.64 (14)
C31—C3—C3A—C9A	179.90 (16)	C9A—N1—C11—C12	−31.2 (2)
C9A—C3A—C4—C4A	0.62 (18)	N2—N1—C11—C12	164.26 (12)
C3—C3A—C4—C4A	175.37 (15)	C9A—N1—C11—C16	148.85 (14)
C9A—C3A—C4—C41	178.75 (12)	N2—N1—C11—C16	−15.73 (19)
C3—C3A—C4—C41	−6.5 (2)	C16—C11—C12—C13	−1.3 (2)
C3A—C4—C4A—C5	−179.09 (13)	N1—C11—C12—C13	178.74 (13)
C41—C4—C4A—C5	2.8 (2)	C11—C12—C13—C14	1.3 (3)
C3A—C4—C4A—C8A	3.01 (18)	C12—C13—C14—C15	0.0 (3)
C41—C4—C4A—C8A	−175.13 (12)	C13—C14—C15—C16	−1.3 (3)
C4—C4A—C5—C6	−177.40 (14)	C14—C15—C16—C11	1.3 (2)
C8A—C4A—C5—C6	0.5 (2)	C12—C11—C16—C15	0.0 (2)
C4A—C5—C6—C7	0.9 (2)	N1—C11—C16—C15	−179.99 (13)
C5—C6—C7—C8	−1.0 (3)	C3A—C4—C41—C42	−64.18 (18)
C6—C7—C8—C8A	−0.4 (3)	C4A—C4—C41—C42	113.86 (15)
C7—C8—C8A—N9	−178.35 (14)	C3A—C4—C41—C46	116.72 (15)
C7—C8—C8A—C4A	1.8 (2)	C4A—C4—C41—C46	−65.24 (18)
C5—C4A—C8A—N9	178.31 (13)	C46—C41—C42—C43	−0.3 (2)
C4—C4A—C8A—N9	−3.7 (2)	C4—C41—C42—C43	−179.45 (14)
C5—C4A—C8A—C8	−1.87 (19)	C41—C42—C43—C44	−0.1 (2)
C4—C4A—C8A—C8	176.15 (13)	C42—C43—C44—C45	0.2 (3)
C8—C8A—N9—C9A	−179.62 (13)	C43—C44—C45—C46	0.1 (2)
C4A—C8A—N9—C9A	0.20 (19)	C44—C45—C46—C41	−0.6 (2)
C8A—N9—C9A—N1	−176.24 (12)	C42—C41—C46—C45	0.7 (2)
C8A—N9—C9A—C3A	4.0 (2)	C4—C41—C46—C45	179.80 (13)
N2—N1—C9A—N9	179.99 (13)		

### Hydrogen-bond geometry (Å, °)

**Table:**

*D*—H···*A*	*D*—H	H···*A*	*D*···*A*	*D*—H···*A*
C12—H12···N9	0.93	2.44	3.0012 (18)	119
C46—H46···N9^i^	0.93	2.52	3.4164 (18)	163
C16—H16···N2	0.93	2.48	2.799 (2)	100

Symmetry codes: (i) −*x*+1, −*y*+1, −*z*.
